# Comment to “Further Considerations on the Research of Parkinson's Disease‐Related Chronic Pain”

**DOI:** 10.1002/mdc3.70372

**Published:** 2025-09-26

**Authors:** Daniel Ciampi de Andrade, Santiago Perez Lloret, Julien Bally, Veit Mylius

**Affiliations:** ^1^ Center for Neuroplasticity and Pain (CNAP), Department of Health Science and Technology, Faculty of Medicine Aalborg University Aalborg Denmark; ^2^ Fundación H. A Barceló Instituto Universitario de Ciencias de la Salud Consejo de Investigaciones Científicas y Técnicas (CONICET) Buenos Aires Argentina; ^3^ Departamento de Fisiología, Facultad de Medicina Universidad de Buenos Aires Buenos Aires Argentina; ^4^ Service of Neurology, Department of Clinical Neurosciences Lausanne University Hospital and University of Lausanne Lausanne Switzerland; ^5^ Department of Neurology Center for Neurorehabilitation Valens Switzerland; ^6^ Department of Neurology Kantonsspital Graubünden Chur Switzerland; ^7^ Department of Neurology Philipps University Marburg Germany

**Keywords:** Parkinson's disease, chronic pain, PD‐related pain, PD‐unrelated pain, nociceptive pain

We are grateful for the interest expressed by Hu in our recent article, *“Distinction and Mutual Influences between Parkinson's Disease‐Related and Unrelated Chronic Pain”* by Hunger, J. et al 2025.^1^ The thoughtful comments highlight important aspects of chronic pain research in Parkinson's disease (PD). We would, however, like to clarify several points raised in the letter.

Hu raises concerns about the representativeness of our Swiss sample of non‐demented PD patients. However, comparisons were drawn. We demonstrated that the clinical presentation of PD‐related pain in Switzerland is not significantly different to that in South America,^2^ but a higher prevalence of PD‐unrelated pain was observed in the Swiss sample.^1^ Translations of PD‐PCS should provide fresh views on how geographically diverse our reports are. We agree that the issue of pain in dementia should be explored further. Since this population often has limited verbal pain reporting, verbal descriptor scales, simple numeric scales or the PAINAD for advanced dementia should be considered.^3^ It was noted that the follow‐up period of our study was relatively short. Compared to most cross‐sectional studies, our validation study already goes a step further by incorporating a follow‐up component to confirm the stability of diagnoses.^2^ Longer‐term follow‐up studies are certainly warranted, but they address different research questions. A further point concerned the reliance on patient self‐reports for pain assessment, and the suggestion to include physiological or neuroimaging measures. It is essential to emphasize that pain, by its very nature, is a subjective experience. Unlike motor assessment, there are no physiological indicators of the pain experience. Tools such as the PD‐PCS, are specifically designed to structure and standardize this subjective reporting. The present approach of subdividing chronic pain into PD‐related and ‐unrelated types before classifying the pain mechanism is based on neurophysiological findings and brain imaging (for review see Tinazzi et al 2025^4^). Correlation analyses involving experimental measures are ongoing and have already been partially implemented.^5^ Hu also expressed that the study could have included more discussion on therapeutic interventions. We agree that the classification of PD‐related pain is only the first step, and that therapeutic research must follow. This process is underway, with clinical trials now investigating tailored therapeutic approaches for different PD‐related pain syndromes (for review see Tinazzi et al 2025^4^). Finally, the letter raises the important issue of psychosocial factors, such as mood disorders and social support, in pain perception. We fully agree that these are integral components of the pain experience. In fact, when we validated the PD‐PCS, we explicitly compared pain categories against classic assessments of mood, quality of life, and sleep, and found meaningful relationships.^2^ This reinforces the view that pain cannot be understood in isolation but must be assessed alongside psychological and social dimensions.

In summary, while we appreciate the constructive remarks of Hu, we respectfully submit that our methodological choices are well grounded. Our study builds upon evidence of cross‐regional comparability, incorporates follow‐up beyond the cross‐sectional norm, applies the gold standard of patient‐reported outcomes, and opens pathways for further therapeutic innovation.

## Authors Roles

1. Research project: A. Conception, B. Organization, C. Execution;
2. Statistical Analysis: A. Design, B. Execution, C. Review and Critique;
3. Manuscript: A. Writing of the first draft, B. Review and Critique.

DCA: 1A, B, C, 2C, 3A, B.

SPL: 1A, B, 2A, B, 3B.

JB: 1C, 2C, 3B.

VM: 1A, B, C, 2C, 3B.

## Disclosures

**Ethical Compliance Statement:** The study protocol was approved by the local Institutional Review Boards in St. Gallen and Lausanne (BASEC: 00502). Written informed consent has been obtained from all participants. We confirm that we have read the Journal's position on issues involved in ethical publication and affirm that this work is consistent with those guidelines.

**Funding Sources and Conflict of Interest:** The study was supported by grants from Parkinson Schweiz, Zambon, and Mundipharma. The authors declare that there are no conflicts of interest relevant to this work.

**Financial Disclosures for the previous 12 months:** DCA is vice chair for the research committee of the European Federation of International Association for the Study of Pain chapters; is section editor for the *European Journal of Pain*; is on the advisory board of *Pain Reports*; declares institutional investigator‐initiated research grants from Novo Nordisk Foundation, Neuroscience Academy Denmark (Lundbeck Foundation), Horizon Europe (Fresco4NoPain European consortium), and the European Research Council. SPLL consulted for Elea Laboratorios (Argentina), has stock options in TeleNeuro Solutions LLC (NJ, USA), and received grants from Agencia de Promoción Científica y Técnica (Argentina). JFB received speaker's honoraries from BIAL, Spirig, Abbvie, Merz, and Medtronic. VM is on the advisory board of *European Journal of Pain*. He declares research grants from Parkinson Switzerland. He received honoraria from Abbvie, Zambon, and BIAL and he consulted for BIAL.

## Data Availability

NA.
